# The effects of human training data (HTD) explanation on purchase intention for artificial intelligence (AI) technologies

**DOI:** 10.1371/journal.pone.0339482

**Published:** 2026-02-02

**Authors:** Stephanie Kwari Dharmaputri, Greg Nyilasy, Anish Nagpal, Jing Lei

**Affiliations:** Department of Management and Marketing, University of Melbourne, Melbourne, Victoria, Australia; Dong-A University College of Business Administration, KOREA, REPUBLIC OF

## Abstract

Consumers exhibit resistance to Artificial Intelligence (AI) technologies despite their widespread deployment. One common thread linking various reasons for this is the perceptions of AI technologies’ lack of inherent human qualities. This paper explores the effect of human training data (HTD) explanation on purchase intention for AI technologies as a potential source-based explainable AI (XAI) solution to this problem. We define HTD explanation as a statement that describes the role that humans play within a given AI system’s training data in lay terms that is easy to understand for end-users. This includes descriptions of what human data is used and for what purpose (e.g., “Our ready-made meals are developed by an AI system trained on millions of people’s food and taste preferences”). We predict HTD explanation to have a positive effect on consumer purchase intention that is mediated by perceived transfer of human essence. We tested our hypotheses in four between-subjects experiments. Study 1 supports the proposed main effect. Studies 2 and 3 show evidence for the mediation mechanism. Study 4 provides marginal evidence that HTD explanation may reduce adoption intention among consumers high pre-existing trust in AI technologies. Our findings suggest that the psychological processes linking HTD explanation to consumer outcomes may be more complex than originally theorized. This study introduces HTD as a form of source-based XAI solution and offers preliminary insight into PET and deep humanization effects, where consumers may perceive AI technologies to possess human qualities by virtue of HTD use.

## Introduction

### Beyond appearances: The limits of surface anthropomorphization in countering AI resistance

The practice of anthropomorphizing, or humanizing, Artificial Intelligence (AI) technologies to improve AI evaluation and purchase intentions has become standard practice in many industries [1–3]. Companies have widely adopted anthropomorphism practices by giving AI technologies a human name, voice, and appearance [3–5]. For example, many smart speakers (e.g., Apple, Google, and Amazon’s smart speakers) are equipped with natural human voice and speech mannerism [1,6]. Samsung’s Neon features lifelike artificial humans designed to serve as virtual receptionists, customer service agents, news reporters, and more [7]. Many AI technologies are also given human names, including Amazon’s Alexa and Samsung’s Sam [3,8,9]. Over the last decade, these humanization efforts have improved AI acceptance among consumers [8]. This positive effect has also been documented by numerous empirical studies highlighting the benefits of AI anthropomorphism efforts on AI acceptance and purchase intention [8–10].

Nevertheless, there are limits to the benefits of anthropomorphism [11]. Despite the general increase in AI acceptance attributed to anthropomorphism efforts, there remains a considerable level of consumer resistance to AI technologies [11,12]. People have previously been documented to exhibit aversion to AI technologies [13,14], including those of anthropomorphized ones [11]. For example, Longoni et al. [14] found that consumers tend to resist medical AI, perceiving it as less capable of understanding individual patient needs. Similarly, Castello et al. [13] showed that consumers are more resistant to AI technologies in subjective domains (e.g., creative or emotional tasks) than in objective domains (e.g., financial advice generation). Further, those who have received assistance from AI technologies (e.g., AI-generated service support, medical advice) would often seek a second opinion from human representatives [15,16]. The degree of AI resistance does vary across contexts. For example, people have been documented to be more resistant to AI tools in hedonic (e.g., indulgent products, sensory experiences) than utilitarian (e.g., health benefits, functionality) domains [17]. People also resist AI solutions more when they are used to make decisions with high stakes (e.g., determining who receives vaccines for a deadly flu variant) compared to those with low stakes (e.g., determining who receives vaccines for a mild flu variant) [18]. Although contextual variation exists, consumer resistance appears to be a consistent issue across domains, underscoring the need to address its underlying drivers.

There are several key reasons for AI resistance, including AI technologies’ perceived opacity, lack of emotions, and inflexibility [18]. First, people perceive AI systems as too opaque, with a “black box” decision logic that is hard to understand [19]. In turn, this leads to mistrust, particularly when outcomes are unexpected or explanations are unclear [19]. Second, AI technologies are often seen as lacking emotional capability [18,20], making them less suitable for tasks perceived as subjective [13]. Third, many believe that AI systems are inflexible, rigid, and lack the adaptability that humans possess [21]. This widespread belief likely arises “...because, historically, machines have operated based on simpler, non-adaptive algorithms that performed only narrow tasks” [18]. This is supported by Longoni et al. [14], who found that a key reason for AI resistance is the perception that AI technologies lack the cognitive flexibility to account for individual uniqueness. Taken together, these factors help explain why consumers continue to resist AI technologies. Overall, while the specific reasons for this residual resistance are widely varied, one common thread among them is the perception that AI technologies lack the inherent qualities that humans have and therefore cannot fully replace humans.

AI resistance persists even when AI technologies are designed to look and sound human [11]. This highlights a gap between perceived appearance and essence, and reveals the limits of current anthropomorphism efforts. This also suggests a deeper theoretical gap: although AI technologies can be designed to appear human, they are still widely viewed as lacking authentic human qualities. This contributes to ongoing AI resistance across domains, marking a notable challenge in need of resolution.

### Human at the core: Human training Data (HTD) as a pathway to reduce AI resistance

Despite advances in humanlike design, AI technologies are still seen to lack authentic human qualities, contributing to ongoing AI resistance. What remains overlooked is that many AI models are built upon data that originates from humans. This form of training data embeds human patterns of thought, judgment, and behavior directly into AI systems through their learning processes. In this way, AI does not merely mimic humans in form. Rather, it also inherits distinctly human qualities from the data on which it is trained. Yet, consumers rarely recognize these deeply embedded human qualities, focusing instead on the mechanical nature of AI technologies.

In this research, we propose that making the human origins of AI training data explicit may help bridge this perceptual gap and reduce resistance to AI technologies. In this regard, we introduce the concept of human training data (HTD). Humans make a major component of AI training data through extensive datapoints generated by their interactions and behaviors. For example, voice assistant AIs are often trained on human speech in the development of their natural language processing capability, allowing them to exhibit humanlike speech and language capabilities [22]. The medical field has observed the use of AI consultation systems trained on physician judgment data to generate advice to patients [23]. Further, some self-driving car AIs are also trained on human driving behavior, which may result in adherence to social norms and even rule-breaking behavior [24]. These examples show that AI systems are often trained on data that reflects how humans think, feel, and behave. We refer to these as human training data (HTD), which is a training dataset that is derived from the cognitive, affective, and behavioral human processes captured within the dataset.

Importantly, HTD originates from humans themselves. As such, AI systems trained on HTD will absorb the human processes embedded within the data and exhibit these through their outputs. This renders AI more human, not by mimicking surface traits or superficial human appearances, but by internalizing the deep psychological processes behind human behavior. Through this, AI systems trained on HTD would inherit the human qualities they are perceived to lack at their core. For example, an HTD-trained medical AI system may inherit the cognitive flexibility of real medical professionals, who often have to take the unique characteristics of each patient into account when forming a diagnosis [23]. As another example, an HTD-trained customer service AI system may possess the emotional intelligence of human service representatives, allowing AI service reps to express empathy to frustrated customers [22].

Yet, there is limited public awareness of the common use of HTD in AI systems. Consumers continue to perceive AI technologies as foreign and lacking human qualities [12]. This lack of awareness may be attributed to the general lack of explainability and transparency regarding the inner workings of AI systems [19,25]. Presently, there is limited explainability on an AI technology’s inner workings, how it operates, and how it was trained [19,25]. This may be traced back to the insufficient communication from technology companies regarding how their AI systems are trained [19,25]. In turn, this has been documented to contribute to the issue of consumer resistance towards AI technologies [25–27]. Ultimately, the disconnect between an AI system’s inner workings and the extent of people’s understanding of it creates a consequential gap that contributes to persistent resistance to AI technologies.

The divide between the actual inner workings of AI systems and the extent to which they are understood by the public presents an intriguing research opportunity. The limitations of surface anthropomorphism strategies that revolve around superficial human qualities (e.g., giving AI technologies a human name, voice, and appearance) call for alternative anthropomorphism strategies that possess the capability to produce perceived humanization that reaches the core of AI technologies, allowing people to reevaluate their pre-existing mental model regarding AI technologies’ lack of inherent human qualities. For that, we propose the provision of HTD explanation to bring these human qualities to light and ultimately shift people’s mental model on these technologies and address the overarching problem of this research.

### The present research

This paper puts forward HTD explanation as a source-based explainable AI (XAI) solution for AI resistance. We address the research question “How does HTD explanation influence consumer purchase intention?” To this, we propose HTD explanation to bring human presence within an AI system’s training dataset to the forefront. Consequently, this highlights the presence of humans at the core of said system. We expect this to result in the perception of symbolic contact between humans and AI technologies, where awareness of human inputs may prompt people to create a meaningful psychological link between humans and AIs. In accordance with the contagion effect, we anticipate this symbolic contact to trigger the process of perceived essence transfer (PET) from human sources to the AI recipient. This process will evoke the perception of AI technologies embodying the essence and immaterial qualities of humans that are captured within the dataset. In turn, we predict this to shift people’s mental model of AI technologies from that of cold unfeeling machines to entities that embody human qualities. We expect this to produce more positive perceptions of AI technologies, leading to higher purchase intention and ultimately reduced AI resistance.

This research makes three key contributions, each with its own nuance. First, we introduce HTD explanation as a source-based XAI strategy and extend explainability research by examining how making the human origins of AI visible can shape consumer perceptions. Second, we offer initial evidence that PET may serve as a promising explanatory mechanism for how consumers make sense of the human qualities embedded in AI technologies. In doing do, our research explores the concept of deep humanization, a perspective on how consumers may perceive AI technologies as fundamentally human-like, not merely through surface-level cues (e.g., voice or appearance), but through perceived exposure to human cognitive, emotional, and behavioral data during development. Third, our moderation findings offer preliminary insight into the heterogeneous effects of HTD explanation. Our findings indicate that HTD explanation may backfire when presented to individuals with high pre-existing trust in AI systems, leading to a decrease in their adoption intention. This underscores the importance of considering individual-level factors in the design and deployment of AI communication strategies.

## Theoretical development

### Human training data (HTD)

Training data refers to the data that is used to construct an AI system, and serves as a core input to AI technologies [28]. It serves as the primary source of information to make predictions, decisions, and other forms of output [29,30]. It also lays the foundation for the intelligence embedded in AI systems, shaping their ability to understand, learn, and adapt to various tasks and challenges.

As previously noted, humans form a major part of AI training datasets. Such datasets act as foundational material upon which AI systems are trained to understand and replicate the thought processes, decisions, and behavioral patterns of humans. We refer to such data as HTD. HTD is routinely used in AI systems across various contexts, including retail, consulting services, and transportation [22,24,31]. To synthesize the scope of HTD applications, Table 1 outlines key uses of HTD across different AI systems.

**Table 1:** HTD applications in AI systems.

Context	Training data	AI system application	Source
General	Speech and conversations – e.g., speech patterns, tone of voice, conversational flow	Customer service chatbots, virtual assistants, transcription, and voiceover AI programs (e.g., eBay, IKEA, FedEx, DHL)	[22]
E-commerce	Online shopping activities – e.g., product clicks, browsing pattern and duration, search queries, purchase history	Product recommender systems (e.g., Amazon, eBay, Target)	[22,31]
E-commerce	Consumer expressed fashion preferences (expressed in surveys, handwritten notes, curated images, and more)	Product recommender systems (e.g., Stitch Fix)	[32]
Transport	Human driving behavior – e.g., following social norms, occasional violation of traffic regulations	Self-driving car (e.g., Waymo, General Motors, Zoox)	[24]
Medical	Physician consultation procedure – e.g., physician conversation with patients, diagnostic procedures	Medical recommender AI systems (e.g., Your. MD, Babylon Health)	[23]
Finance	Financial expert judgments – e.g., past expert assessments on potential market share growth, stock susceptibility to disruption, and other expert judgments	Investment recommender system (e.g., Wealthfront, Robinhood)	[33]
Medical	Surgeon hand movements	Minimally invasive surgical robots (e.g., Intuitive Surgical, Medtronic)	[34,35]
Retail	Human preferences data	AI-produced product lines (e.g., Coca Cola, Unilever)	[36]

Table 1 summarizes HTD applications across different domains and AI systems. While we focus on HTD as a general feature of AI systems, we recognize that human training data may take on different forms. To this, we put forward three different types of human training data: cognitive, affective, and behavioral. We propose this definition and typology based on the tripartite model commonly used in psychology and neuroscience (e.g., [37,38]). This classification is often used to break down complex human processes into distinct categories for better understanding and has similarly influenced the marketing field. In consideration of the human origins of the training data under focus, we opted to apply the tripartite model for classifying different types of human training data.

We define cognitive human training data as data that is derived from human thinking processes. Cognition refers to the mental activity of information processing [39]. It encompasses various processes including memory, pattern identification, perception, problem solving, and mental imagery [40]. In line with this literature definition, we propose cognitive human training data to encompass data on human judgments, decision logic, problem solving process, and information processing. In accordance with the psychology literature on cognition [39,40], we propose that cognitive human training data would contain information on how humans make decisions, interpret situations, weigh risks, and rank their priorities. The integration of human decision logic and mental models within a given cognitive human data means that humans and AI systems will share decision logic of the same nature. Koren et al. [23] offer an illustrative example of cognitive HTD, where a medical AI consultant is trained on data containing rich information on the judgment, decision logic, and problem-solving processes used by medical professionals.

We define affective human training data as data that is derived from human affect and emotions. Affect is defined as the experience of feelings and emotions [41]. Emotion is defined as a strong affective reaction tied to a specific cause, including a specific event, object, person, or experience [42]. This includes both positive and negative emotional states ranging from happiness, excitement, sadness, anger, fear, and more [41]. In line with this, we define affective human training data as data that is derived from human affect and emotions, including both positive and negative emotional states. Many customer service bots that are trained using affective data are equipped with sentiment analysis capabilities, and possess the ability to identify customer emotions and respond appropriately to these emotional cues [43]. In turn, this informs AI technologies of what emotions to display in response to certain situations [44], while data on tones of voice have been used to inform AI technologies on what tone to adopt to convey certain emotions [45].

Finally, we define behavioral HTD as data that is derived from human actions and behavior. Behavior is defined as tangible physical actions that are observable to others [46]. This includes data on physical movements and actions that humans make when interacting with their surroundings [46], e.g., walking [47], hand and legs movements when interacting with objects and surrounding environment [34,48,49] Behavioral training data serve as a bridge between AI systems and human actions. Behavioral training data provides AI systems with information and understanding into the nuanced ways in which humans interact with their surroundings. This allows AI systems to mimic human behavior and produce behaviors that are akin to human behavior. The insights derived from this training dataset also provides AI systems with information on how they need to respond to certain events in a manner that is appropriate to the human audience.

These different HTD types provide AI systems with an understanding of the nuanced ways in which humans interact with their surroundings, and may be used in training AI systems on how to behave in a humanlike manner. We now summarize these three distinct types of HTD in Table 2.

**Table 2:** Human training data types.

Cognitive	Affective	Behavioral
Derived from human thinking processes	Derived from human affect and emotions	Derived from human actions and behavior
Encompasses data on human judgments, decision logic, problem solving process, and information processing. Allows AI systems to emulate human thought processes and make decisions that align closely with those made by humans.	Includes both positive and negative emotional states, as well as mood and affectivity May be used to inform AI systems how to assess and respond to certain scenarios expected to generate an emotional reaction.	Provides AI systems with information on how humans interact with their surroundings. Allows AI systems to mimic human behavior
Example(s): Medical AI trained on physicians’ diagnostic processes [23], Investment recommender system trained on financial expert judgments [33]	Example(s): Affectiva – monitors driver emotional states to improve road safety [50], Customer service bots with sentiment analysis and the ability to identify emotional cues [43]	Example(s): Minimally invasive surgery AI robots [34], Housekeeper robots [48]

### HTD explanation

AI systems are often built on human-derived data, including human data of cognitive, affective, or behavioral nature. However, consumers often remain unaware of these human foundations. This lack of awareness reinforces the perception that AI systems are purely mechanical and devoid of human influence, which contributes to the issue of persisting AI resistance. Given this, making the human foundations of AI systems more visible is a promising way to explain how AI systems operate, while lifting resistance. This may be achieved through the provision of HTD explanation to end-users.

We define HTD explanation as the provision of a statement describing the role that humans play within a given AI system’s training source. This statement clarifies the origin and nature of the human data used to train the system, using language that is understandable to everyday users. For example, an AI-developed ready-made meals might be accompanied by the explanation: “These ready-made meals are developed by an AI system trained on millions of people’s food and taste preferences.” Similarly, an HTD explanation of how a self-driving car functions may read: “This car’s AI is trained on how millions of real drivers would react in similar situations.”

We propose that consumer response to the HTD explanation would be generally positive. As reasoned earlier, consumers show persistent resistance to AI technologies due to the perceptions of these technologies being lacking in inherently human qualities [11,13,14,18]. Yet, many of these technologies are fundamentally shaped by human data – a detail that many people overlook. By making the human contribution to AI more salient, HTD explanation may prompt consumers to reconsider their assumptions about AI, leading to more favorable perceptions and greater purchase intention for such technologies. Formally:

**H1**: HTD explanation positively influences purchase intention.

### Perceived essence transfer (PET)

We anticipate HTD explanation to increase purchase intention through the mediating mechanism of perceived essence transfer (PET), grounded in contagion theory [51,52]. By highlighting the human origins of an AI system’s training data, HTD explanation brings human presence to the forefront. For example, the statement “this AI system uses customer taste and preferences data in developing our company’s ready-made meals” highlights the human sources (i.e., customers) and their input (i.e., taste and preferences) explicitly. This explicit link to human input may prompt consumers to infer that the AI system has absorbed the essence of human food preferences captured its training data. As a result, we anticipate this to shift consumers’ fundamental perceptions of AI systems from that of a foreign machine with no understanding of human food preferences to one that embodies human understanding of food preferences.

This whole effect can be explained through the principles of contagion. The contagion effect documents the phenomenon of PET between a source and a recipient that encounter each other [53]. It posits that immaterial qualities or the “essence” of a source can be transferred to a recipient through contact [54]. Specifically, it proposes that “...people, objects, and so forth that come into contact with each other may influence each other through the transfer of some or all of their properties” [53, p159]. These core qualities or “essence” are considered analogous to a soul or personal energy, which in this case is perceived to be transmitted from one source to a recipient [53]. PET effects have been observed to occur between human sources and objects [52,55], including clothing [56,57], personal belongings [58], artworks [59], and more. For example, people have been found to believe that the essence of an artist is captured in their artworks [59]. Possessions of celebrities have also been documented to fetch high prices due to the belief that their essence is contained in their personal belongings [58].

The contagion effect is characterized by three primary characteristics: permanence, holographic properties, and dose insensitivity [52]. The permanence principle asserts that perceived contact, however brief, will lead to the perception of enduring essence transfer from the source to the recipient [60]. This principle encompasses contacts that are considered both direct and indirect [52,55]. Direct contact concerns physical touch between two parties (e.g., a handshake, a hug), while indirect contact concerns symbolic contact between two parties (e.g., shared use of object) [55]. According to the permanence principle, once essence transfer through direct or indirect contact is perceived to occur, the recipient is regarded to be resistant to attempts of purification [52]. In other words, the recipient is perceived to be permanently changed following the contact [52,60]. The holographic principle posits that a small quantity of essence embodies most, if not all, of the characteristics of its source [60,61]. Finally, the dose insensitivity principle suggests that a minimal amount of essence transfer is sufficient to produce an all-encompassing perception of transfer of the source’s immaterial qualities and properties into the recipient [60]. Contagion operates in an all-or-nothing manner, in which only minimal contact between the source and recipient is required to produce a significant contagion effect, with prolonged contact generating only marginal additional contagion effect [52].

### PET and AI technologies

In the case of AI technologies, what makes PET unique is that the contact between human and AI is symbolic, not physical. AI systems trained on human data never directly interact with their sources in a physical manner. Although AI systems never physically touch their human sources, symbolic contact has been documented to produce PET effects as effectively. Prior work shows that essence beliefs arise even from indirect traces such as photographs or handwriting [53,54]. In the case of AI, human data traces are not mere proxies but direct imprints of people’s thoughts, emotions, and behaviors, making them especially potent carriers of perceived essence. Unlike contagion effects observed with tangible objects such as clothing or artworks, the human essence is perceived to be transferred through large-scale data traces that represent human thoughts, emotions, and behaviors. The integration of human cognitive, emotional, and behavioral inputs creates a psychologically meaningful link between humans and the AI system. We expect HTD explanation to increase the salience of this connection, resulting in perceived symbolic contact between the two parties. In turn, we expect this to trigger contagion-based inferences, whereby human-like qualities are believed to be absorbed into the AI technology.

Another distinctive feature of the present context is that both human input and AI system are immaterial. Yet, their interaction can be understood through the same logic as material contagion. Just as essence is believed to transfer through physical contact between objects, symbolic contact between two immaterial entities (such as human data and AI systems) may trigger PET perceptions where human essence is believed to be transferred into the AI system. In both cases the process is the same: the essence of one entity is believed to be passed onto the other, whether through physical or symbolic contact. In sum, what makes PET unique in the AI context is that the “contact” is symbolic rather than physical. The scale of contact is vast, as millions of human datapoints may be seen as embedding diverse aspects of human essence into the system. Second, the form of contact is immaterial-to-immaterial, as human data and AI systems both lack physical substance. Together, these features distinguish PET in the AI domain from prior contagion phenomena.

Following perceived symbolic contact, contagion-based PET inferences will begin. The present case involves a no-contact, symbolic transfer between two immaterial entities: human data and AI systems. Human data operates as direct imprints of thoughts, emotions, and preferences. When an AI system is trained on such data, observers may perceive the system as having absorbed human essence, even without physical interaction. In line with contagion principles, this symbolic transfer is expected to be viewed as enduring (permanence), with even a small quantity of human input seen as embodying the whole (holographic), and any amount of transfer treated as sufficient to fully transmit the source’s essence (dose insensitivity) [60,61]. As a result, observers may come to perceive AI systems not as cold and impersonal machines, but as technologies that embody human qualities. By shifting these fundamental mental models, HTD explanation and PET processes may reduce consumer resistance to AI and increase willingness to adopt AI-enabled products and services. Formally:

**H2**: HTD explanation positively influences purchase intention through the mediating effect of perceived transfer of human essence.

**Fig 1:**
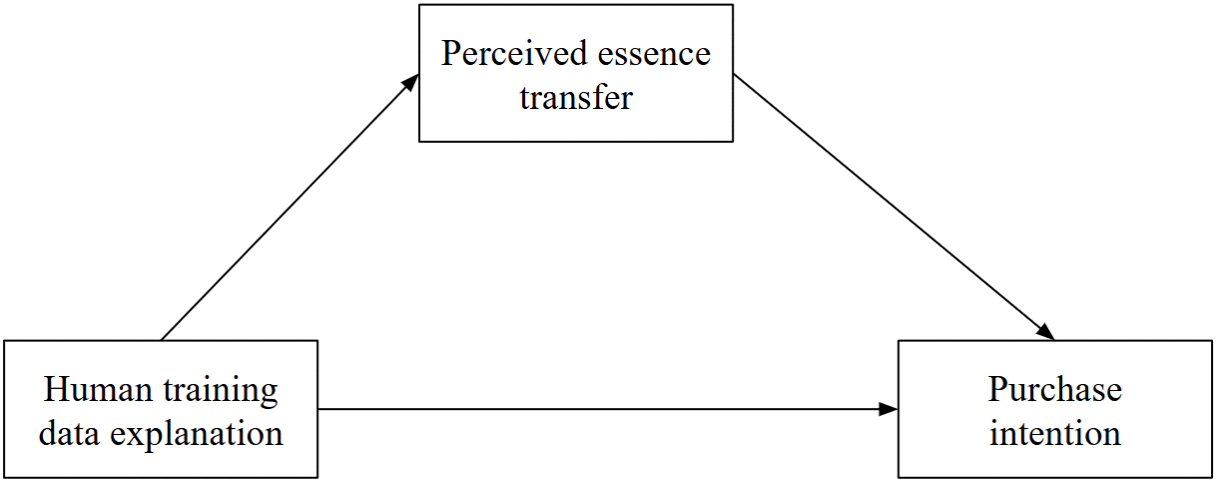
Conceptual framework.

Our conceptual framework is illustrated by Fig 1:

## Methods

The hypotheses were tested with a series of between-subjects experiments. Participants across studies were given a scenario depicting an AI technology trained on HTD (vs control) prior to being asked to evaluate the AI technology they read about. Studies 1 through to 3 test the main effect and mediation hypotheses in the contexts of a potential car crash and ready-made meals. Study 4 was then added to test a potential moderation mechanism in an aged-care services context. Data collection for this project began on 17 November 2022 and ended on 26 October 2023. Study participants provided written consent, and no minors were recruited for the study. This project was approved by the University of Melbourne Ethics Committee. Informed consent was obtained by presenting participants with a series of statements describing the nature of the research, including study purpose, procedures, and participants rights (e.g., voluntary participation and the ability to withdraw at any time). Participants were asked to indicate their understanding of these statements and to provide consent by choosing “yes” or “no” before proceeding with the study.

### Study 1: The effect of HTD explanation on purchase intention in car crash scenario

#### Procedures.

Study 1 investigates the main effect of HTD explanation on purchase intention (H1). This study also explores the role of PET in mediating the relationship between HTD explanation and purchase intention (H2). Study participants were recruited through Prolific to participate in an online experiment (*n* = 197). This study had two experimental conditions that participants were randomly assigned to: HTD vs Control condition. Two participants did not pass the attention check, in which study participants had to answer “Disagree” to one of our questionnaire items, leaving a total of 195 usable responses (51.8% male, *M*_*age*_=37.09, *SD*_*age*_=14.50).

Study participants were given a scenario depicting a potential car crash situation involving an AI self-driving car. Participants were asked to imagine crossing the road when they see a self-driving car driving straight into their direction. Participants assigned to the HTD condition were given the information that the AI system within the car is trained on how millions of real human drivers would react in the same situation. Participants assigned to the control condition were not given any information on how the AI system was trained. More details on the experimental procedure can be found in S1 Appendix. After reading the scenario, participants were asked to complete a questionnaire with the following measures:

The dependent variable, purchase intention was measured on a 7-point Likert scale (1 = “Strongly disagree”, 7 = “Strongly agree”) using a scale that is adapted and expanded from Cordell, Wongtada and Kieschnick [62]. The scale items include: “I would be likely to buy a self-driving car with this AI system,” “I would be inclined to buy a self-driving car with this AI system,” and “I would be willing to buy a self-driving car with this AI system.”

To measure the mediating mechanism, PET, we adapted the “inclusion of self” scale as a visual-based operationalization of perceived transfer of human essence to AI technologies (Aron, Aron and Smollan [63]). Study participants were asked “To what extent do you believe that human essence is integrated into the AI system depicted in the scenario?”. Study participants were then presented with seven pairs of circles with different degrees of overlap; ranging from barely touching to almost completely overlapping. In each pair, one circle was given the label “Human”, and the other given the label “AI”. We asked study participants to select one of the seven pairs of circles to represent their belief as to how much human essence is integrated within the AI system. The greater the overlap is, the greater the perceived integration of human essence in the AI system depicted in the scenario. To control for order effect, we also counterbalanced the orders in which the seven circles appear to participants. Half of study participants saw the seven pairs of circles in the following order: Lowest overlap – Greatest overlap. The other half were presented the seven pairs of circles in the following order: Greatest overlap – Lowest overlap.

As an alternative outcome, capturing if respondents exhibited a change in functional quality perceptions we measured “perceived likelihood of stopping,” by a single question: “How likely do you think this self-driving car in the scenario will stop last minute and avoid hitting you?” (0 = “Will hit me”, 100 = “Will not hit me”). Through the inclusion of this variable, we sought to rule out an alternative explanation that HTD explanation would increase purchase intention by altering quality perceptions (i.e., the car’s ability to act safely) rather than through PET (i.e., our proposed mechanism).

As controls, we measured study participants’ prior attitude towards AI technologies on a 7-point semantic differential scale with bipolar anchors (Dislike – Like, Negative – Positive, Unfavorable – Favorable) as well as demographics (age, gender, first language).

We also included a manipulation check question, in which study participants were asked how the AI system depicted in the scenario was trained (“Trained on how millions of real human drivers would react” vs “No information was given”).

#### Results.

Logistic regression results indicate that the manipulation was successful with 91.8% correct classification (*p* < .001, *B* = 2.491). The Cronbach’s alpha scores indicate that all scales have good reliability (Purchase intention *α*= .971; Attitude *α*= .976).

**Fig 2:**
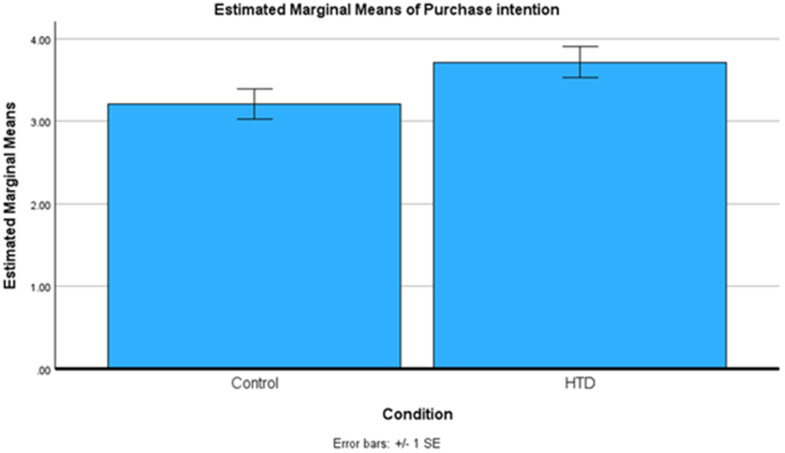
Study 1 means plot.

We conducted an independent-samples t-test to investigate the effect of HTD explanation on purchase intention (H1). We found that HTD explanation has a significant positive effect on purchase intention, in which purchase intention is higher in the HTD condition relative to the control group, *t*(193) = 1.912, *p* = .029, *M*_*HTD*_ = 3.72, *SD*_*HTD*_ = 1.95, *M*_*control*_= 3.21, *SD*_*control*_= 1.77, *Cohen’s d* = .274). These results are also summarized in Fig 2.

We conducted a mediation analysis using Process Hayes Model 4 to test the role of PET in mediating the relationship between HTD explanation and purchase intention (H2). We decided to use Process Hayes to test our hypotheses as this analysis method allows us to test our predictions with bias-corrected bootstrapping, which is superior to earlier approaches such as the Baron and Kenny method [64]. Our analysis uses 5,000 bootstrap samples and a 95% confidence interval. The analysis results reveal a non-significant mediation effect, 95% CI [LLCI = −.0217, ULCI = .1406]). The direct effect of HTD explanation on purchase intention is marginally significant (*p* = .088; *B* = .221). The effect of HTD explanation on PET is non-significant (*p* = .187). The effect of PET on purchase intention is significant (*p* = .000, *B* = .286).

For robustness, we re-ran our main analysis while controlling for covariates. We first ran correlations to investigate potential covariates to the hypothesized relationship between HTD explanation and purchase intention. Specifically, we examined whether purchase intention is significantly correlated with gender, age, language, and pre-existing attitudes toward AI systems. We only included two genders (male and female) in the analysis, as the small number of participants in other categories (e.g., non-binary, prefer not to say) limited the feasibility of including them in the analysis. Out of the four variables, two variables are significantly correlated with purchase intention: pre-existing attitude (*r* = .664) and gender (*r* = −.161). We therefore included them as covariates in subsequent analysis. We ran a univariate ANOVA using purchase intention as the dependent variable, HTD explanation as the independent variable, and pre-existing attitude towards AI systems and gender as covariates. The effect of pre-existing attitude towards AI systems on purchase intention is significant (*p* < .001, partial *η²* = .435). The effect of gender on purchase intention is not significant (*p* = .117). The effect HTD explanation on purchase intention is still significant with the covariate controlled for (*p* = .006, *F* = 7.667, partial *η²* = .040. Participants who were given HTD explanation are more likely to express higher purchase intention relative to those in control condition.

We also conducted an independent-samples t-test to investigate the effect of HTD explanation on our exploratory dependent variable (perceived likelihood of stopping). The R1assumption of equal variances was met (*p* = .923; *F* = .009). We found that HTD explanation has a non-significant effect on perceived likelihood of stopping (*p* = .396, *M*_*HTD*_ = 68.95, *SD*_*HTD*_ = 24.46, *M*_*control*_= 68.02, *SD*_*control*_= 24.33, *Cohen’s d* = .038), and that there is no difference in perceived likelihood of stopping between the HTD and control groups.

#### Discussion.

Study 1 results provide support for our main effect hypothesis (H1), in which HTD explanation has a significant positive effect on purchase intention. This effect remains significant after covariate effects are controlled. However, the mediation hypothesis (H2) is not supported. In addition, we found no significant effects of HTD explanation on the variable perceived likelihood of stopping. This removes the alternative explanation that the results may be explained by differences in underlying product quality perceptions between the HTD and control groups. In Study 2, we sought to replicate Study 1 results with a larger sample size to reexamine the mediation hypothesis.

### Study 2: The effect of HTD explanation on PET and purchase intention in car crash scenario

#### Procedures.

Study 2 seeks to replicate Study 1 results with a larger sample size and tests for our mediation (H2) hypotheses. Study participants were recruited through Prolific to participate in an online experiment (*n* = 302). This study had two experimental conditions that participants were randomly assigned to: HTD vs Control condition. Four participants did not pass the attention check question, leaving a total of 298 usable responses (50.0% male, *M*_*age*_=40.29, *SD*_*age*_=15.28).

Study participants were given the same materials and measures as in Study 1, in which they were shown a scenario depicting a potential car crash situation involving an AI self-driving car. More details on the experimental procedure can be found in S1 Appendix. In this study, we also included an exploratory outcome variable for negative affect, fear, measured using a scale adapted from Van Zomeren [65] on a 7-point Likert scale (1 = “Strongly disagree”, 7 = “Strongly agree”). The following items were included in the scale: “This self-driving car is frightening,” “I found this self-driving car to be threatening,” “I was afraid of this self-driving car,” “This self-driving car unsettled me.” Past research revealed that people experience negative emotions and become emotionally afraid when asked about their general attitude towards self-driving cars [66]. As our scenario depicts a potential collision event, we decided to include this additional dependent variable to rule out that our results may be explained by differences in negative affect rather than our proposed mediation mechanism of PET. Finally, we included a manipulation check question as in Study 1.

#### Results.

Logistic regression results again indicate that the manipulation was successful with 88.4% correct classification (*p* < .001, *B* = 2.066). The Cronbach’s alpha scores indicate that all scales have good reliability (Purchase intention *α*= .945; Fear *α*= .940; Attitude *α*= .967).

**Fig 3:**
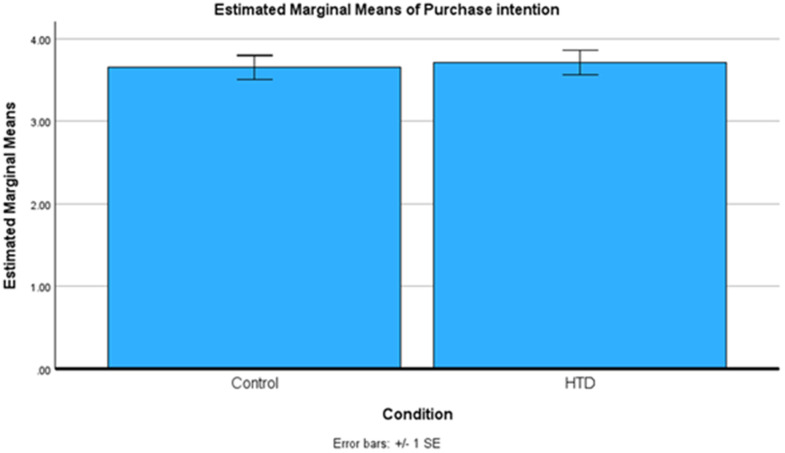
Study 2 means plot.

We conducted an independent-samples t-test to investigate the effect of HTD explanation on purchase intention. The assumption of equal variances was not met, therefore the t-test conducted were heteroskedastic (*p* = .028; *F* = 4.849). We found that HTD explanation has a non-significant effect on purchase intention (*p* = .392, *M*_*HTD*_ = 3.71, *SD*_*HTD*_ = 1.87, *M*_*control*_= 3.66, *SD*_*control*_= 1.67, *Cohen’s d* = .032). The results indicate that there is no difference in purchase intentions between the HTD and control groups. These results are also visualized in Fig 3.

We conducted a mediation analysis using Process Hayes Model 4 to test the role of PET in mediating the relationship between HTD explanation and purchase intention (H2). Our analysis uses 5,000 bootstrap samples and a 95% confidence interval. The analysis results reveal a significant mediation effect, *B* = .076, *SE* = .039, 95% CI [LLCI = .0018, ULCI = .1546]). The direct effect of HTD explanation on purchase intention is non-significant (*p* = .622). The effect of HTD explanation on PET is significant (*p* = .05; *B* = .212). The effect of PET on purchase intention is significant (*p* = .000, *B* = .358).

For robustness, we re-ran our main analysis while controlling for covariates. We found three variables that are significantly correlated with purchase intention: gender (*r* = −.179), age (*r* = .228), pre-existing attitude towards AI systems (*r* = .606). Based on our correlations results, we ran a univariate ANOVA using purchase intention as the dependent variable, HTD explanation as the independent variable, and the following covariates: gender, age, pre-existing attitude towards AI systems. There were no significant effects of on purchase intention gender (*p* = .144). The effect of pre-existing attitude towards AI systems on purchase intention is significant (*p* = < .001, partial *η²* = .333). The effect of age on purchase intention is significant (*p* = .01, partial *η²* = .023). The main effect of HTD explanation on purchase intention remains non-significant (*p* = .294). Next, we re-ran our mediation analysis with covariates included. Our analysis uses 5,000 bootstrap samples and a 95% confidence interval. Pre-existing attitude has a significant effect on PET (*p* = .000, *B* = .427) and purchase intention (*p* = .000, *B* = .520). Gender has a marginally significant effect on PET (*p* = .058, *B* = .383) and purchase intention (*p* = .064, *B* = −.304). Age has a non-significant effect on PET (*p* = .291) and significant effect on purchase intention (*p* = .005, *B* = .015). The mediation analysis reveals significant full mediation effect when the covariate is controlled for, *B* = .039, *SE* = .020, 95% CI [LLCI = .0057, ULCI = .0826]. As in our main analysis, the direct effect of HTD explanation on purchase intention is non-significant (*p* = .632). The effect of HTD explanation on PET is significant (*p* = .016; *B* = .239). The effect of PET on purchase intention is significant (*p* = .001, *B* = .162).

Finally, we found a non-significant effect of HTD explanation on fear (*p* = .129, *M*_*HTD*_ = 4.23, *SD*_*HTD*_ = 1.62, *M*_*control*_= 4.02, *SD*_*control*_= 1.60, *Cohen’s d* = .131), with the assumption of equal variances met (*p* = .759; *F* = .094). The results indicate that there is no difference in fear between the HTD and control groups.

#### Discussion.

Study 2 provides support for our mediation hypothesis (H2), in which HTD explanation positively influences purchase intention through PET. This mediation effect still stands when covariate effects are controlled. In saying this, we note that the effect size for the mediation mechanism is small. Our additional analysis on fear yielded non-significant results. This removes the alternative explanation that our results may be explained by differences in negative affect between the HTD and control groups. However, our results indicate that HTD explanation does not have a direct effect on purchase intention. Given the mixed evidence from the two studies, we test our hypotheses within a new experimental context of AI-developed ready-made meals in Study 3. This new experimental context was chosen to address potential elements in the self-driving car scenario (e.g., risk, danger) that may have influenced our findings and contributed to the observed mixed results. Additionally, this context was also chosen to provide greater variation in experimental settings and control for potential product category effects by shifting to a low-risk, everyday consumer good.

### Study 3: The effect of HTD explanation on PET and purchase intention in ready-made meals scenario

#### Procedures.

Study 3 investigates the mediation mechanism (H2) in a new research context, in which study participants were given a scenario depicting AI-developed ready-made meals. We recruited marketing undergraduate students to participate in our online pretest in exchange for course credit (*n* = 300). We incorporated a student sample in this study to increase sample diversity across studies. Additionally, given the low-risk context of fast-moving consumer goods, students represent a fitting demographic due to their frequent engagement with such products, hence increasing ecological validity. This study had two conditions that participants were randomly assigned to: HTD vs Control condition. Twenty-seven participants had either withdrawn or did not pass the attention check question, leaving a total of 273 usable responses (40.7% male, *M*_*age*_= 19.75, *SD*_*age*_= 4.13).

Study participants were given a scenario involving AI-created ready-made meals. Study participants were asked to imagine shopping for ready-made meals at the grocery store. They were informed that as they browsed through the available options, they learned that the ready-made meals are developed by an AI system. Participants who were randomly assigned to the HTD condition were given the information that the AI system used to create the meals are trained on human data and were informed that this data is based on millions of people’s food and taste preferences. Participants assigned to the control condition were given no information on how the AI system was trained. For detailed information on experimental stimuli, please refer to S2 Appendix. After reading the scenario, participants were asked to complete a questionnaire with the following measures: purchase intention, PET and prior attitude (measured as in Studies 1 and 2). The following demographic information was also collected: gender, age, language.

As a manipulation check, we included a multiple-choice question, “Please write briefly about your understanding of how the AI system in this particular scenario was trained. What did the scenario say the AI system was trained on?” The two choices given for this question were “No information was given in regard to how the AI system was trained” and “The AI system is trained on millions of people’s food and taste preferences.” We used the same attention check measure as in our previous studies.

#### Results.

Logistic regression results indicate that the manipulation was successful with 77.2% correct classification (*p* < .001, *B* = 1.554). The purchase intention scale has good reliability (Purchase intention *α*= .906).

**Fig 4:**
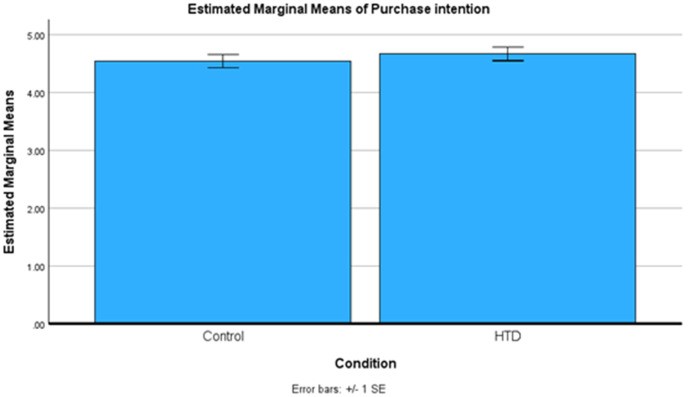
Study 3 means plot.

We conducted an independent-samples t-test to investigate the effect of HTD explanation on purchase intention. The assumption of equal variances was met (*p* = .495; *F* = .467). We found that HTD explanation had a non-significant effect on purchase intention (*p* = .220, *M*_*HTD*_ = 4.67, *SD*_*HTD*_ = 1.31, *M*_*control*_= 4.54, *SD*_*control*_= 1.40, *Cohen’s d* = .094). The results indicate that there is no difference in purchase intentions between the HTD and control groups. These results are also summarized in Fig 4.

We conducted a mediation analysis using Process Hayes Model 4 to test our mediation hypothesis (H2). Our analysis uses 5,000 bootstrap samples and a 95% confidence interval. The analysis results reveal a significant full mediation effect, *B* = .047, *SE* = .025, 95% CI [LLCI = .0066, ULCI = .1066]. The direct effect of HTD explanation on purchase intention is non-significant (*p* = .839). The effect of HTD explanation on PET is significant (*p* = .012; *B* = .238). The effect of PET on purchase intention is significant (*p* = .000, *B* = .198).

For robustness, we re-ran our main analysis while controlling for covariates. We ran correlations to investigate covariates as in previous studies. We found two variables that are significantly correlated with purchase intention: gender (*r* = −.134), pre-existing attitude towards AI systems (*r* = .389). These two variables were then used in our subsequent analysis as covariates. We ran a univariate ANOVA using purchase intention as dependent variable, HTD explanation as the independent variable, and the following covariates: gender, pre-existing attitude towards AI systems. The effect of gender on purchase intention is non-significant (*p* = .107). The effect of pre-existing attitude towards AI systems on purchase intention is significant (*p* = < .001, *F* = 3.595, partial *η²* = .210). The effect HTD explanation on purchase intention remains non-significant (*p* = .375). Next, we also re-ran our mediation analysis (5,000 bootstrap samples and a 95% confidence interval) with pre-existing attitude towards AI systems included as a covariate. Gender was excluded as a covariate due to its non-significant effect in prior analysis. Pre-existing attitude has a significant effect on PET (*p* = .000, *B* = .335) and purchase intention (*p* = .000, *B* = .381). Our analysis results indicate that the mediation effect weakens and becomes marginal when controlling for covariate effects, *B* = .029, *SE* = .019, 95% CI [LLCI = −.0002, ULCI = .0725]. The direct effect of HTD explanation on purchase intention is non-significant (*p* = .481). The effect of HTD explanation on PET is significant (*p* = .006; *B* = .253). The effect of PET on purchase intention is significant (*p* = .024, *B* = .114).

#### Discussion.

Our Study 3 results provide support for H2, in which HTD explanation positively influences purchase intention through PET. The mediation effect is significant without covariates, though the effect size is small. This effect is also attenuated and becomes marginally significant when controlling for pre-existing attitude towards AI systems. While the indirect effect becomes marginal when we incorporated control variables in the model, the individual pathways through PET remain significant in both models.

The mixed findings on the direct effect of HTD explanation on purchase intention in our studies suggest that there are potential moderators that may strengthen or weaken the main effect relationship. We explore this possibility by investigating a potential moderation mechanism at play in Study 4.

We identified pre-existing trust in AI technology to be a potential moderator of HTD explanation’s effect on purchase intention. Pre-existing consumer trust in technology can be viewed as a prior conviction that is reached through a cognitive evaluation process [12]. Trust in technology is marked by consumer confidence and predisposition to rely on said technology despite any uncertainty and potential losses [12]. Trust in AI technologies can be characterized by one’s willingness to accept its output, such as its recommendations and decisions [67]. It reflects one’s confidence in the capability, competence, and reliability of the technology [68]. Past research has repeatedly demonstrated that trust plays an important role in the adoption and acceptance of new technologies [2,10,69], including AI systems and products [70].

We expect HTD explanation to be particularly important and relevant to those with *low pre-existing trust* in AI technologies. We previously highlighted that one reason for people’s continued resistance to AI technologies stems from: 1) the belief that these technologies lack inherently human qualities [11,18], and; 2) the lack of explainability regarding the inner workings of AI technologies [19,25]. To this, HTD explanation serves to: 1) facilitate the perceptions of perceived transfer of human essence into AI technologies, and; 2) improve the explainability of an AI technology’s inner workings. As HTD explanation addresses the underlying reasons for people’s resistance towards AI technologies, we expect the positive effect of HTD explanation to be greater for individuals with low pre-existing trust in AI technologies.

Conversely, we predict the positive effects of HTD explanation on adoption intention to be reversed among consumers with high pre-existing trust in AI technologies. We note that some individuals may hold high levels of pre-existing trust for AI technologies. This may be attributed to their positive experience with AI technologies, and previous successful interactions with such technologies [67,68]. As such, high-trust consumers typically maintain a stable mental model in which AI is reliable, accurate, and dependable. With a strong baseline of favorable perceptions [68], high-trust consumers typically see little need for further justification and are inclined to accept AI output without additional explanations.

In this context, HTD explanation may disrupt rather than reinforce confidence. High-trust consumers experience little need for additional justification, and readily accept AI outputs as trustworthy. In this state, an added explanation can be interpreted as unnecessary elaboration. Moreover, HTD explanation aims to shift people’s mental models of the inner workings of AI systems by emphasizing its human foundation. By making the system’s human data sources salient, it may alter how consumers conceptualize how AI works, guiding their understanding of its decision-making processes. We predict that such reframing can subtly unsettle the favorable baseline that high trust consumers already have. By casting the technology in a different light, HTD explanation may introduce dissonance into an otherwise consistent belief set. Rather than reassuring, this added information can act as a diagnostic cue that something warrants concern, prompting heightened scrutiny and resistance. This aligns with prior work suggesting that those with high confidence in their convictions may respond negatively to information that unsettles their prior beliefs [71,72]. Thus, for high-trust consumers, HTD explanation may ultimately reduce adoption intention. We test this moderation hypothesis within an aged care context in Study 4, using adoption intention as the dependent variable. The formal hypothesis is:

**H3**: Pre-existing trust in AI technologies moderates the relationship between HTD explanation and adoption intention, such that the positive effect of HTD explanation on adoption intention is boosted (reversed) among individuals with low (high) levels of pre-existing trust in AI systems.

### Study 4: Pre-existing trust as a moderator in effect of HTD explanation on adoption intention in aged care scenario

#### Procedures.

This study investigates the role of pre-existing trust in moderating the relationship between HTD explanation and adoption intention of AI technology (H3) in an aged care scenario. This new context was chosen to provide greater variation in experimental settings and to control for potential product category effects, consistent with the rationale behind Study 3. Study participants were recruited through Prolific to participate in an online experiment (*n* = 387). As before, this study had two experimental conditions that participants were randomly assigned to: HTD vs Control condition. Eleven participants did not pass the attention check question, leaving a total of 376 usable responses (58.5% male, *M*_*age*_=36.15, *SD*_*age*_=13.11).

Study participants were asked to read a scenario in which they must secure an aged care home for their aging parents. Study participants were informed that the aged care home they found employs AI companion robots as part of their support staff. The scenario outlines the types of companionship provided by the companion robots, including engaging in small talk, providing emotional support, and providing general companionship in the client’s daily activities. Those in HTD condition were informed that the AI companion robots are trained on how real aged care workers perform their duties. Those in the control condition were informed that the AI companion robots are trained on best aged care practices. For more details on the experimental stimuli, please refer to S4 Appendix. Following this, study participants were asked to indicate their adoption intention of the companion robots’ services for their parents. After reading the scenario, participants were asked to complete a questionnaire with the following measures: We measured the dependent variable, adoption intention using a scale adapted from Venkatesh, Thong and Xu [73] on a 7-point Likert scale (1 = “Strongly disagree”, 7 = “Strongly agree”). The following items included in the scale: “I would possibly use this AI robot’s companion service for my parents in the future,” “I would possibly try to use this AI robot’s companion service for my parents,” “I would potentially plan to use this AI robot’s companion service for my parents.” Companion robots employed by aged care homes as part of support staff may not be available to purchase for personal ownership; however, clients may have varying levels of adoption intention of the companion robots’ services for their aging parents, and can either choose to adopt the technology during their parents’ stay at the aged care home or forgo the option. This provides the basis for our choice of dependent variable in this study.

As a moderator, we measured pre-existing trust in AI, using a 9-point multi-item scale adapted from Sirdeshmukh, Singh and Sabol [74] with the anchors: “Very undependable” – “Very dependable,” “Very incompetent” – “Very competent,” “Of very low integrity” – “Of very high integrity,” “Very unresponsive to its users” – “Very responsive to its users”. Additionally, the following demographic information was also collected: gender, age, language.

We also measured perceived empathy, measured using five items from Iglesias et al. [75] and Jung et al. [76]: “This AI companion robot would understand my parents’ needs”, “This AI companion robot would understand my parents’ feelings,” “This AI companion robot would care about my parents’ mental health”, “This AI companion robot would focus on providing the best services to my parents,” and “I would feel comfortable having this AI companion robot interact with my parents.” All items were measured using a 7-point Likert scale (1 = “Strongly disagree, 7 = “Strongly agree”).

We included two attention check questions, in which study participants had to select the answers “Somewhat Disagree” and “Agree” for two separate questions. For manipulation check purposes, we included a multiple-choice question, “Please share briefly about your understanding of how the AI system in this particular scenario was trained. What did the scenario say the AI was trained on?” The two choices given for this question were: 1) Real human aged care workers, or 2) Best aged care practices.

#### Results.

Logistic regression results indicate that the manipulation was successful with 93.4% correct classification (*p* < .001, *B* = −2.642). The Cronbach’s alpha scores indicate good reliability for all scales (Adoption intention *α*= .975; Trust *α*= .884).

**Fig 5:**
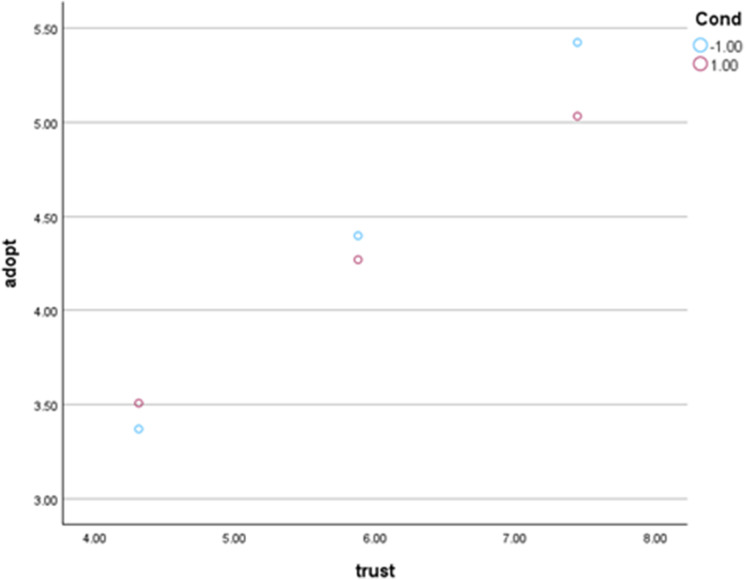
Study 4 Johnson-Neyman moderation plot.

We conducted a moderation analysis using Process Hayes Model 1 to test our moderation hypothesis (H3). Our analysis uses 5,000 bootstrap samples and a 95% confidence interval. The analysis results reveal a marginally significant negative moderation effect (*p* = .08; *B* = −.085). A Johnson-Neyman analysis revealed that the effect of HTD explanation on purchase intention was significant and negative when baseline trust in AI technologies was greater than 8.24 (measured on a 9-point semantic differential scale), *B* = −.264, *SE* = .135, *p* = .05, 95% CI [−.530,.002]. This suggests that for consumers with high prior trust in AI, HTD explanation may backfire and reduce their intention to purchase AI-generated products. The Johnson-Neyman moderation plot is given in Fig 5.

For robustness, we re-ran our main analysis while controlling for covariates. We ran correlations to investigate covariates as in previous studies. We found two variables that are significantly correlated with adoption intention: perceived empathy (*r* = .778) and gender (*r* = .106) and thus we used them as covariates (age and language are not significantly correlated with adoption intention). We re-ran our moderation analysis with covariates included. Our analysis uses 5,000 bootstrap samples and a 95% confidence interval. Perceived empathy has a significant effect on adoption intention (*p* = .000; *B* = .936). Gender has a non-significant effect on adoption intention (*p* = .754) The interaction between HTD explanation and pre-existing trust in AI systems on adoption intention is marginally significant and negative with the covariate controlled for (*p* = .071; *B* = −.068). Probing the interaction, our Johnson-Neyman output reveals that the effect of HTD explanation on purchase intention is marginally significant and positive for people with low pre-existing trust in AI technologies. Specifically, the interaction is marginally significant and positive for baseline trust levels of 1.00 (*p* = .078, *B* = .341, *SE* = .193, 95% CI [−.038,.792]) to 2.90 (*p* = .094, *B* = .212, SE = .126, 95% CI [−.037,.460]). Although marginal, these findings suggest that HTD explanation does improve adoption intention for individuals with low pre-existing trust in AI technologies when individual differences are controlled for.

#### Discussion.

Our results provide marginal support for our moderation hypothesis (H3), in which pre-existing trust in AI systems moderates the effect of HTD explanation on adoption intention. Our findings indicate that HTD explanation has a marginal effect in boosting (reversing) the positive effects of HTD explanation on adoption intention for individuals with low (high) pre-existing trust in AI technologies. These findings present important implications for researchers and practitioners considering using HTD explanation as a means to improve AI adoption or purchase intentions. Our study results are summarized in Table 3. We now close this section and discuss our findings.

**Table 3:** Summary of study results*.*

Study	Context	Data Source	Mediator (M)/ Moderator (W)	Results	Conclusion
Study 1	Self-driving car/Car crash scenario	Prolific (*n*=197; attn check =195); Age (*M* = 37.09, SD=14.50); 51.8% male	M: PET	Purchase intention (Sig.), *t*(193) = 1.912, *p* = .029, *M*_*HTD*_ = 3.72, *SD*_*HTD*_ = 1.95, *M*_*control*_ = 3.21, *SD*_*control*_ = 1.77, *Cohen’s d* = .274 Mediation – Purchase intention, Process Model 4 (N.S.), LLCI = -.0217, ULCI = .1406	H1 supported H2 not supported
Study 2	Self-driving car/Car crash scenario	Prolific (*n*=302; attn check = 298); Age (*M* = 40.29, SD=15.28); 50.0% male	M: PET	Purchase intention (N.S.), *p* = .392 Mediation – Purchase intentions, Process Model 4 (Sig.), *B* = .076, *SE* = .039, 95% CI [LLCI = .0018, ULCI = .1546]).	H1 not supported H2 supported
Study 3	Ready-made meals	First-year marketing undergraduate students (*n* = 300; attn check = 273); Age (*M* = 19.75, SD=4.13); 40.7% male	M: PET	Purchase intention (N.S.), *p* = .220 Mediation – Process Model 4 (Sig.), *B* = .047, *SE* = .025, 95% CI [LLCI = .0066, ULCI = .1066	H1 not supported H2 supported
Study 4	Aged care companion robots	Prolific (*n* = 387; attn check = 376) Age (*M* = 36.15; *SD* = 13.11); 58.5% male	W: Pre-existing trust in AI technologies	Moderation (Marginal Sig.), *p* = .08; *B* = -.085	H3 supported (marginal)

## General discussion

This paper aims to address the research question: “How does HTD explanation influence consumer purchase intention?” This research is motivated by consumers’ resistance to AI technologies stemming from these technologies’ perceived lack of inherent human qualities. We highlighted that many AI technologies are trained on HTD, though consumers are often unaware of this. This presents an opportunity to: 1) reduce AI resistance, and; 2) improve purchase or adoption intention by presenting consumers with HTD explanation. We predicted HTD explanation to result in higher purchase intention through PET. We also predict pre-existing trust in AI technologies to moderate this, such that the positive effect of HTD explanation is greater (reversed) for individuals with lower (higher) pre-existing trust in AI technologies.

Our study findings provide partial support for our hypotheses and reveal important nuances in how consumers respond to HTD explanation. While Study 1 showed a main effect of HTD explanation on purchase intention, this effect was not consistently observed across studies. Studies 2 and 3 provide partial support for our mediation hypothesis (H2), demonstrating early evidence of HTD explanation operating through PET mechanism, consistent with contagion theory [51,52]. This provides initial support to the notion that emphasizing the human origin of AI systems may trigger inferences about AI systems inheriting human qualities. At the same time, we note the mixed replication pattern and the relatively small observed mediation effect sizes. Taken together, the relatively small mediation effect observed suggests that PET’s influence may be attenuated or reversed by boundary conditions, hence its limited effects on shaping purchase intentions.

To investigate the inconsistencies in our findings, Study 4 examines the role of pre-existing trust in AI technologies as a potential moderator to the relationship between HTD explanation and adoption intention (H3). Study 4 provides some support for our moderation hypothesis. This study reveals a marginally significant and positive effects of HTD explanation on adoption intention for individuals with low pre-existing trust in AI technologies when covariates are controlled. We also find this effect to be reversed among people with high pre-existing trust in AI technologies. Given the marginal significance, these findings should be regarded as tentative and exploratory. However, our findings provide initial insights into HTD explanation’s non-uniform effect, where it may produce both positive and negative reactions depending on the audience. This pattern aligns with past research showing that explainability is not always perceived positively, and may trigger reactance depending on user characteristics [77,78]. This also aligns with Munoz and Rucker [71], who found that individuals with high confidence in their convictions may respond negatively to new, unfamiliar information that disrupts their cognitive prior. Although the interaction effect was marginally significant, it offers a plausible explanation for the mixed main effects observed in earlier studies and highlights the importance of consumer heterogeneity in response to explainability strategies.

Another potential moderating factor that may have contributed to this twofold effect is the human sources used in the data. Humans are diverse creatures and may be seen as either virtuous or flawed. Depending on whether the human source is perceived positively or negatively, the perceived transfer of their essence may either produce positive or negative contagion. Evidently, past research has also established that PET does not always yield positive results, such as when the human source is known to be a moral transgressor or criminal [55,56]. Given the vast number of humans and the wide spectrum of their characteristics in the data set, some study participants may interpret their PET positively, while others may interpret it negatively. These contrasting views on the favorability of this essence transfer may counterbalance each other, leading to minimal means difference in purchase or purchase intentions between the HTD and control group, while still providing some support to our mediation mechanism.

Our findings provide some support to our predictions that HTD explanation may serve useful in shifting people’s fundamental perceptions of AI technologies from that of a pure automaton to a technology that possesses the capability to embody human qualities. Given the variability in findings, we note that these conclusions remain preliminary, and future research should be undertaken. We now discuss the theoretical and managerial implications of these findings.

### Theoretical implications

This paper presents three key contributions. First, we introduce and conceptualize HTD as a psychologically meaningful feature of AI systems that may shape consumer response to AI technologies. While training data is foundational to AI development, prior work has predominantly examined it from technical or computational perspectives. We extend this conversation into the consumer domain by proposing HTD as an explainable characteristic that can be communicated to end-users and shape perceptions of AI technologies. In doing so, we also introduce HTD explanation as a form of source-based XAI, one that emphasizes the human origins of an AI system’s training data rather than its inner workings or decision logic. In turn, this offers a way to address core concerns around AI’s perceived lack of humanness by making its human foundations more visible. While the empirical support is preliminary, this study offers a starting point for future work at the intersection of AI explainability and consumer behavior.

Second, we extend the concept of PET to the domain of human-AI interaction. Drawing from the contagion literature [51,52], we suggest that consumers may perceive a symbolic transfer of human essence into AI systems when informed that the system was trained on human data. While this psychological process has previously been examined in the context of physical objects and interpersonal interactions [52,53], we apply it here to a non-physical, data-driven context. Our findings offer initial empirical support for PET as a potential mechanism through which consumers interpret AI systems as possessing humanlike qualities.

Building on this mechanism, we explore what may be described as deep humanization effects. Through PET, consumers may build the perception that an AI system has internalized core human qualities, such as thought, emotion, or judgment, through its training process. This form of humanization reflects an attribution of humanness that is internalized, grounded in the belief that human essence has transferred into the technology. By contrast, surface humanization arises from more superficial perceptual cues, such as the presence of a human face, voice, or conversational style [3–5]. These cues can make an AI appear human-like, but they do not alter consumers’ inferences about the system’s underlying essence of being machine-like. This distinction also reflects different psychological routes of humanization. Surface humanization is driven by external resemblance and often triggers heuristic judgments based on appearance or behavior. In contrast, deep humanization is driven by causal inferences about the human origins of AI training data. In this research, we found initial evidence of consumers reasoning that if AI has been trained on human data, it must carry forward human essence that is transferred into the AI recipient. While surface humanization may shape perceptions of human-likeness, deep humanization suggests a more profound reappraisal of what an AI system is, implying that the technology may embody qualities once thought to be uniquely human. While our evidence is preliminary, it raises the possibility that perceptions of humanness can emerge not only from how AI looks or behaves, but also from how it is made. Overall, our findings suggest that PET may serve as a promising but context-dependent mechanism through which HTD explanation influences consumer perception by creating deep humanization effects. These preliminary findings also add nuance to existing work on anthropomorphism by highlighting that humanization effects are multifaceted, and can operate at different levels of psychological depth.

Third, we present early evidence that consumer responses to HTD explanation may vary depending on pre-existing trust in AI technologies. The effects of HTD explanation on adoption intention appear to be marginally positive for individuals with low pre-existing trust in AI technologies. This suggests that HTD explanation does have some efficacy to lower AI resistance and increase adoption intentions for individuals who hold negative baseline perceptions towards these technologies. However, our results show that HTD explanation could backfire for individuals with very high trust in AI, potentially lowering their intention to adopt AI-generated products. While the interaction effects warrant further investigation, it raises the possibility that explainability strategies are not universally effective and may be sensitive to individual differences. This underscores the importance of considering audience characteristics when designing communication about AI systems.

### Managerial implications

The presence of PET as an underlying mechanism indicates that managers have an opportunity to strategically emphasize the human origins of AI training data to shape the perceptions of a deeply humanized AI technology among consumers. However, the downstream effects on purchase and adoption intentions appear to be context-dependent and limited across consumer groups. While our research provides some evidence for HTD explanation’s efficacy in creating deep humanization effects via PET, its net impact on behavior-level outcomes is limited, potentially due to mixed reactions from consumers.

This variation in consumer interpretation means that the practical benefits of HTD explanation may be conditional. Our findings provide tentative evidence that HTD explanation could resonate more with consumers who hold lower baseline trust in AI technologies, while it may have negative impact among those with higher pre-existing trust in AI technologies. Our results highlight the importance of audience segmentation and cautious message tailoring when experimenting with HTD-based communication strategies in AI-driven product contexts. Managers seeking to make use of this strategy need to pilot test HTD messaging in segmented campaigns. This may entail running controlled tests where different versions of HTD explanations (alongside a condition without HTD explanation) are presented across audience segments. Following this, managers may then compare consumer response across segments, including their click-through rates, purchase rates, product trial uptake, and other behavioral outcomes of interest. Pilot tests may also incorporate different framings of HTD explanation. This may entail providing variations in information on the expertise, diversity, or representativeness of the human data sources to identify which angle produces the most positive outcomes. This recommendation offers managers a set of actionable steps to experiment with HTD explanation in practice while also creating opportunities to capitalize on it if a certain framing proves effective.

### Societal and ethical implications

Beyond theoretical and managerial outcomes, our findings point to societal implications for how AI technologies are perceived and adopted. We set out to examine whether HTD explanation could offer a solution to the ongoing problem of consumer resistance toward AI technologies. However, what we found instead is a more complex picture. Specifically, we found public reactions to HTD explanations to be non-uniform and may depend on individuals’ differences (e.g., differences in pre-existing trust in AI technologies). This variation points to a broader societal tension: while increasing awareness about AI’s human underpinnings could help reduce AI resistance in some segments, it may simultaneously disrupt favorable perceptions in others. In practical terms, this implies that HTD explanation could shape public discourse and perceptions of AI technologies in uneven ways. As AI technologies continue to permeate everyday life, understanding how people interpret the humanization of AI systems becomes an important societal concern, warranting further research to explore these complexities. For policymakers, this underscores the need to carefully test and frame human-data narratives, ensuring that communication strategies do not unintentionally polarize public attitudes toward AI technologies. For example, if governments introduce public-sector AI (e.g., welfare eligibility tools) and emphasize that these systems are trained on citizen data, such messaging may be received differently across audiences, with no guarantee of uniform acceptance. This suggests that policy communication about HTD use should proceed cautiously, with small-scale trials used to assess reactions before broader implementation.

Our findings also connect to ongoing debates in AI ethics. Ethical debates on transparency and explainability include concerns about AI’s data sources. For managers, this means that deploying HTD explanation is not only a communication choice but also an ethical one. For example, describing an aged care companion robot as trained on real human aged care workers may enhance transparency and the explainability of this technology. However, it may also raise the ethical dilemma of whether companies should disclose information that they know could divide audiences. The tension here lies between two principles: on one hand, transparency is a core tenet of XAI and ethical AI practices [19,25]; on the other, disclosure that triggers resistance in some groups may undermine the broader goal of building public trust in AI technologies. More than that, firms may become unwilling to disclose such information altogether, which could reduce transparency and work against broader calls for explainable AI. For policymakers, these findings underscore the importance of developing guidance on when and how firms should disclose the use of human training data through HTD explanation, ensuring that disclosure practices build trust without creating new forms of resistance.

### Limitations and future research

We now note our study limitations and direction for future research. First, our studies relied on self-reported measures of purchase or adoption intention, which may not always translate into actual behavior. While intention is a widely used proxy in consumer research, we acknowledge the absence of behavioral measures or field experiments as a key limitation in our paper. This limitation highlights the need for future studies to incorporate behavioral measures or field experiments to test ecological validity. This limitation also reflects broader constraints in developing field-ready, controlled HTD manipulations. Embedding experimentally varied HTD explanations into various AI systems would require backend access, systematic control over explanation delivery, and the ability to track downstream consumer behavior. These conditions make it technically and logistically challenging to run such studies. In saying this, we acknowledge that a field study would help validate the ecological relevance of our findings. Provided the necessary infrastructure is available to researchers, future research should incorporate behavioral outcomes or field experiments. These experiments should assess whether the psychological effects of HTD explanation extend to real-world consumer choices and purchase behavior, thereby establishing the ecological validity of our framework.

Second, given the small mediation effects we observed, future research may examine factors that may have attenuated the PET effect in the current studies. As discussed earlier, these may include the presence of moderators such as prior trust, which was then examined in Study 4. Another potential moderator relates to the variability in how human sources are perceived by users. Differences in perceived source favorability may lead participants to interpret the essence transfer as either positive or negative, potentially balancing out the overall effect. Future studies could build on these insights by manipulating these moderating factors to assess their influence on the strength and direction of the PET pathway.

Third, given the non-uniform effects of HTD explanation, future studies would also benefit from including additional control variables that may shape these responses. This may include controlling for prior AI exposure, AI usage experience, technological literacy and knowledge, and other cultural factors that may contribute to individual differences. Future research could also explore how variations in the perceived nature, quality, diversity, or credibility of HTD source moderate consumer response to HTD explanation. This exploration may reveal additional factors that may have played a role in the observed inconsistencies in our findings. Additionally, as the moderation effect of prior trust was marginal, future research could validate the stability of this effect by increasing sample size or adjusting the HTD disclosure method (e.g., altering its framing, length, or level of detail) to test whether the effect generalizes across different presentation formats, thereby strengthening the preliminary nature of the current conclusions.

Fourth, we also recommend testing our conceptual framework in other contexts. One factor that may have contributed to our present findings is the depiction of uncommon technologies in our experimental scenarios. While the media has increased consumers’ knowledge of self-driving cars, this technology is still not commonplace and is still going through many trials in the present. As such, study participants may struggle to picture themselves purchasing or using these technologies regardless of the training data used, hence contributing to the small means difference observed in our studies. Future research may use AI technologies that are more commonplace for further testing. This includes customer service chatbots, virtual assistants, and smart home devices. These technologies are widely available and accepted, providing a more relatable foundation for the future experiments.

Next, we recruited participants primarily through online panels and, in one study, through a university student sample. While useful for early-stage theory testing, the generalizability of these findings to broader consumer populations remains limited. Future work should replicate and extend our study using more diverse samples to test the robustness of the effects. In particular, we recommend broadening demographic and cultural diversity in forthcoming studies. Future work should employ more diverse and representative samples, incorporate behavioral and field experiments, and systematically account for these boundary conditions to strengthen the robustness of the findings.

Finally, while this paper focuses on HTD as a general feature of AI systems, human training data can take on different forms, e.g., cognitive, affective, or behavioral HTD. Future research may explore whether these HTD types have additive or distinctive effects on purchase and adoption intentions depending on the AI context. For example, affective data may have a stronger impact in high-emotion-demand contexts (e.g., mental health support AI tools). Cognitive HTD may be more influential in decision-driven or analytical domains (e.g., strategic planning, managerial decision support systems). Behavioral HTD may be seen more positively in action-oriented contexts that rely on human performance or behavioral data (e.g., fitness coaching, physical rehabilitation guidance). Exploring these nuances would deepen understanding of how different facets of human data shape AI perceptions and consumer responses.

## Conclusions

This research examined the effects of HTD explanation on purchase intention for AI technologies. We predicted a positive effect of HTD on purchase intention, mediated by PET. Our findings provide some support to our hypotheses, though the inconsistent nature of our findings may indicate that the effect is context dependent and needs further investigations. We found both the direct and indirect effects of HTD explanation on purchase intention, though inconsistent and with relatively weak effect sizes. Further, we found that the effect of HTD explanation on the outcome variable is marginally moderated by pre-existing trust in AI technologies. This provides initial evidence for the existence of boundary conditions that may further specify this effect.

This research presents several exploratory contributions. First, it conceptualizes HTD as a potential form of source-based XAI rooted in the perceived human origins of AI systems, shifting the focus from algorithmic transparency to source-based humanization. Second, we explore the broader construct of deep humanization through the lens of contagion and PET. In doing so, our research tentatively points to psychological mechanisms that may help explain how consumers attribute human-like qualities to AI technologies. Finally, our research identifies pre-existing trust in AI technologies as a moderating factor that determines how consumers will respond to HTD explanation. To this, our findings highlight that individual characteristics could play a role in shaping the effectiveness of HTD explanations. Our key study limitations include the reliance on self-reported intentions, and the lack of control for contextual factors. We suggest future research to examine variations in HTD explanations and HTD types, incorporate relevant control variables, and test the framework in ecologically valid settings using behavioral outcomes and field studies. Overall, this study provides an early step toward understanding how revealing the human foundations of AI technologies can impact consumer responses, laying important groundwork for future research.

## Supporting information

S1 Appendix Studies 1 and 2 Experimental Stimuli. (DOCX)

S2 Appendix Study 3 Experimental Stimuli. (DOCX)

S3 Appendix Study 4 Experimental Stimuli. (DOCX)

S4 Appendix Study 5 Experimental Stimuli. (DOCX)
