# Supplementary material for: The effects of human training data (HTD) explanation on purchase intention for artificial intelligence (AI) technologies
Source: PLoS One. 2026 Feb 2;21(2):e0339482. doi: 10.1371/journal.pone.0339482 (PMC12863500; doi:10.1371/journal.pone.0339482)
Supplement: S1 Appendix — (DOCX) [file pone.0339482.s001.docx]

**S1 Appendix. Studies 1 and 2 Experimental Stimuli.**

***Appendix S1.1: Human training data condition***

Imagine the following scenario.

 
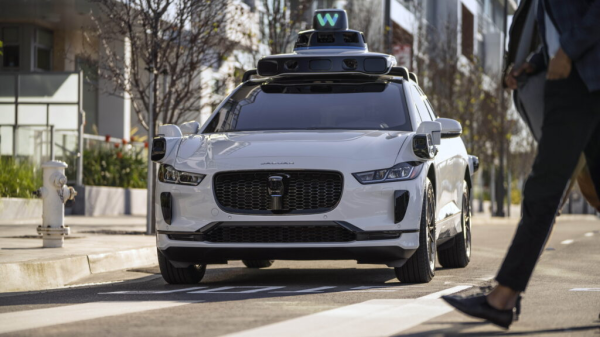


Imagine you are crossing a street.

All of a sudden, you saw a self-driving car controlled by AI driving in your direction. You had missed the car when you were crossing and have just seen it now.

Keep in mind that AI systems in cars like this are **trained on how millions of real human drivers would react in situations like this.**

***Appendix S1.2: Control condition***

Imagine the following scenario.

 
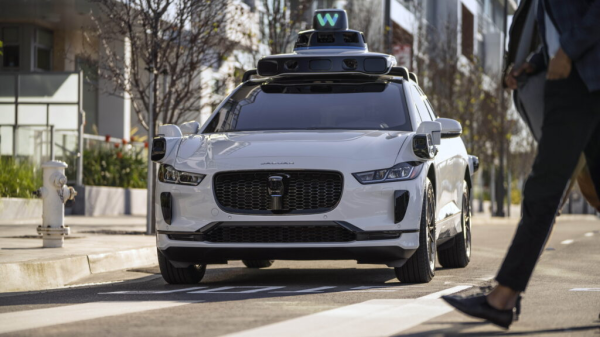


Imagine you are crossing a street.

All of a sudden, you saw a self-driving car controlled by AI driving in your direction. You had missed the car when you were crossing and have just seen it now.
