# Supplementary material for: The effects of human training data (HTD) explanation on purchase intention for artificial intelligence (AI) technologies
Source: PLoS One. 2026 Feb 2;21(2):e0339482. doi: 10.1371/journal.pone.0339482 (PMC12863500; doi:10.1371/journal.pone.0339482)
Supplement: S2 Appendix — (DOCX) [file pone.0339482.s002.docx]

**S2 Appendix. Study 3 Experimental Stimuli.**

***Appendix S2.1: Human training data condition***

Imagine the following scenario.

  
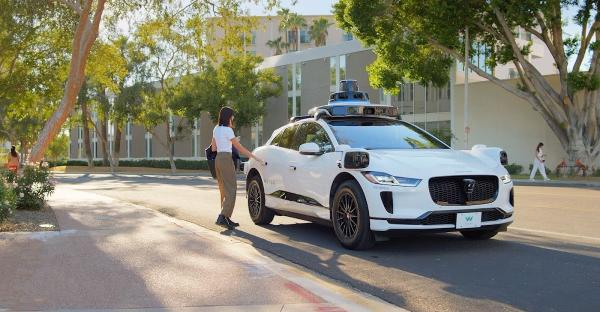


You are looking to book a car through a ride-hailing service (i.e., a service like Uber).

The ride service assigns **a self-driving car** controlled by an **artificial intelligence (AI) system** for your trip.

The ride service asks you to confirm your understanding of potential risks associated with self-driving cars before finalising your booking.

For your knowledge, AI systems in self-driving cars are **trained on how millions of real human drivers would normally drive.**

***Appendix S2.2: Control condition***

Imagine the following scenario.


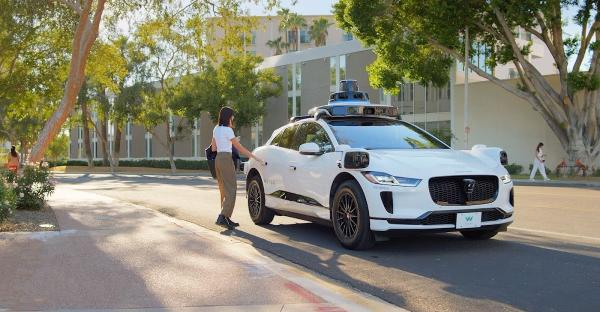


You are looking to book a car through a ride-hailing service (i.e., a service like Uber).

The ride service assigns **a self-driving car** controlled by an **artificial intelligence (AI) system** for your trip.

The ride service asks you to confirm your understanding of potential risks associated with self-driving cars before finalising your booking.
