# Supplementary material for: The effects of human training data (HTD) explanation on purchase intention for artificial intelligence (AI) technologies
Source: PLoS One. 2026 Feb 2;21(2):e0339482. doi: 10.1371/journal.pone.0339482 (PMC12863500; doi:10.1371/journal.pone.0339482)
Supplement: S3 Appendix — (DOCX) [file pone.0339482.s003.docx]

**S3 Appendix. Study 4 Experimental Stimuli.**

***Appendix S3.1: Human training data condition***

Imagine the following scenario.

  
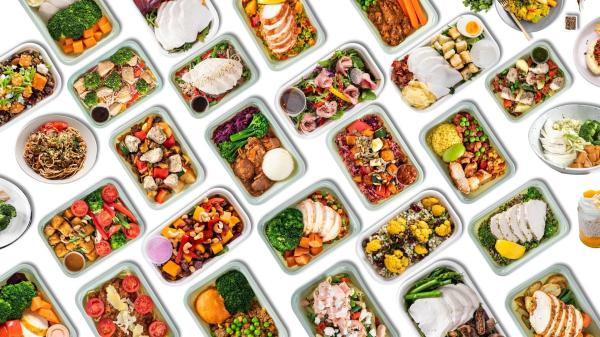


You are looking to purchase ready made meals at the grocery store. As you browse through the available options, you learn that they are developed by an **artificial intelligence (AI) system.**

The AI system used to create the ready made meals are trained on **human data**. Specifically, it is **trained on millions of people's food and taste preferences**.

***Appendix S3.2: Control condition***

Imagine the following scenario.

  
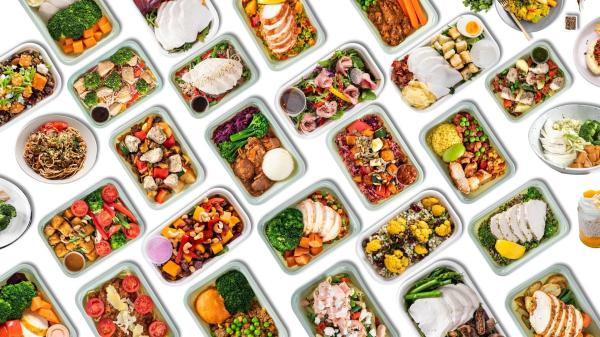


You are looking to purchase ready made meals at the grocery store. As you browse through the available options, you learn that they are developed by an **artificial intelligence (AI) system.**
