# Supplementary material for: The effects of human training data (HTD) explanation on purchase intention for artificial intelligence (AI) technologies
Source: PLoS One. 2026 Feb 2;21(2):e0339482. doi: 10.1371/journal.pone.0339482 (PMC12863500; doi:10.1371/journal.pone.0339482)
Supplement: S4 Appendix — (DOCX) [file pone.0339482.s004.docx]

**S4 Appendix. Study 5 Experimental Stimuli.**

***Appendix S4.1: Human training data condition***

Imagine the following scenario.

Your parents are growing older and you have discussed available options with them to ensure that they get the best care and comfortable retirement years. You have come to an agreement to have them cared for in an aged care home. As part of the care provided, this new type of home has artificial intelligence (AI) companion robots.

The types of companionship and support these robots provide include:

- Initiating social interaction, engaging in small talk, chatting about common interests and current event
- Providing emotional support - offering encouragement, listening to anxieties and concerns
- Providing general companionship in the client’s day-to-day activities (e.g., watching movies together)
- Keeping track of overall well-being - asking "How are you?", mood tracking, sleep quality tracking

Importantly,

These companion robots are **trained using data on how real human aged care workers perform their duties.**

***Appendix S4.2: Control condition***

Imagine the following scenario.

Your parents are growing older and you have discussed available options with them to ensure that they get the best care and comfortable retirement years. You have come to an agreement to have them cared for in an aged care home. As part of the care provided, this new type of home has artificial intelligence (AI) companion robots.

The types of companionship and support these robots provide include:

- Initiating social interaction, engaging in small talk, chatting about common interests and current event
- Providing emotional support - offering encouragement, listening to anxieties and concerns
- Providing general companionship in the client’s day-to-day activities (e.g., watching movies together)
- Keeping track of overall well-being - asking "How are you?", mood tracking, sleep quality tracking

Importantly,

These companion robots are **programmed based on best practice aged care protocols**.
